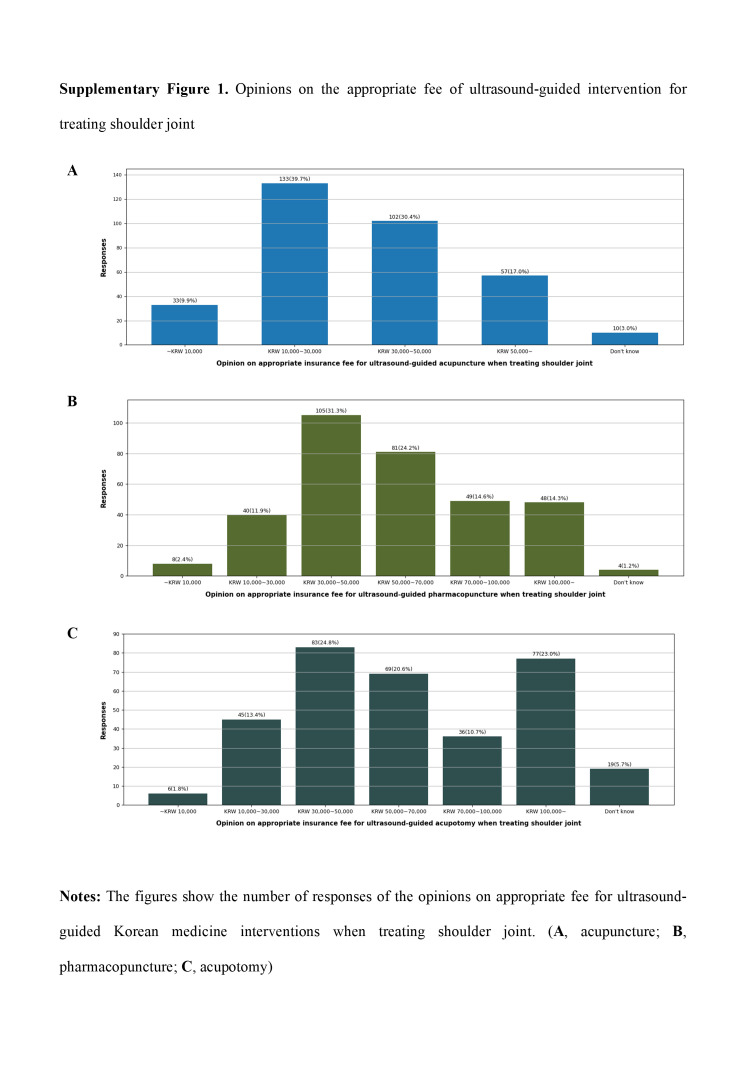# Survey on the current usage of ultrasound-guided procedures in Korean Medicine Clinics and Hospitals

**DOI:** 10.1097/MD.0000000000037659

**Published:** 2024-04-05

**Authors:** Ju Yeon Kim, Jung Min Yun, Sook-Hyun Lee, Yoon Jae Lee, Dong Kun Ko, In Heo, Woo-Chul Shin, Jae-Heung Cho, Byung-Kwan Seo, In-Hyuk Ha

**Affiliations:** a Jaseng Spine and Joint Research Institute, Jaseng Medical Foundation, Seoul, Republic of Korea; b Korean Medical Imaging Association, Jayeonsaeng Korean Medicine Clinic, Yongin-si, Republic of Korea; c School of Korean Medicine, Pusan National University, Yangsan, Republic of Korea; d Department of Korean Medicine Rehabilitation, Kyung Hee University Medical Center, Seoul, Republic of Korea; e Department of Rehabilitation Medicine of Korean Medicine, Kyung Hee University, Seoul, Republic of Korea; f Department of Acupuncture & Moxibustion, College of Korean medicine, Kyung Hee University Hospital at Gangdong, Kyung Hee University, Seoul, Republic of Korea.

**Keywords:** acupotomy, acupuncture, Korean medicine, pharmacopuncture, ultrasound-guidance

## Abstract

Ultrasound-guidance is applied during the administration of Korean medicine (KM) interventions to improve the safety and effectiveness of the interventions. Although many case series and reports on the use of ultrasound-guided KM interventions have been reported, no study has investigated the current use of ultrasound-guidance in clinical practice by doctors of Korean medicine (KMDs). An online survey was conducted with questions examining the status of ultrasound-guidance usage among KMDs practicing in various KM clinical settings. Survey responses from 335 KMDs were collected. Ultrasound started to be widely used in the clinical practice of KM since 2022. The primary objective of using ultrasound-guidance was “To improve the accuracy and efficacy” by 54.6%. Ultrasound-guidance was most frequently applied for shoulder joint diseases, and pharmacopuncture was the most frequently used intervention (76.1% and 90.4%, respectively). The respondents reported that effectiveness could be enhanced the most in nerve entrapment syndromes and especially when used in shoulder joints. Over 90% of KMDs responded that the safety and efficacy of treatment, specialty, and patients’ satisfaction were improved after adopting ultrasound-guidance. Moreover, 94.9% of KMDs agreed with the necessity for reimbursement of ultrasound-guidance in KM under national health insurance coverage. Most KMDs responded that they had positive perceptions regarding the clinical use of ultrasound-guidance in KM in terms of treatment effects, safety, and patient satisfaction, and the need for national health insurance coverage of the service. Our findings may provide practice-based evidence for conducting clinical studies.

## 1. Introduction

Ultrasound-guidance has been applied in a range of areas in clinical medicine to improve safety and obtain the desired effect when using invasive procedures, such as nerve blocks, catheterization, and biopsy.^[1–4]^ Similarly, to achieve the same goals, ultrasound-guidance has been applied in traditional Korean medicine (KM) when procedures, such as acupuncture, pharmacopuncture, and acupotomy, are administered on acupuncture points.^[5]^

Visual observation of the surrounding anatomical structures with ultrasound may contribute to the safe practice of acupuncture procedures.^[6]^ In this regard, a previous study developed a standard operating procedure for ultrasound-guided acupuncture with 44 acupuncture points in high-risk areas on the chest, abdomen, neck, and shoulder.^[7]^ In addition to the safety aspect, clinical studies have reported that ultrasound-guided interventions were more effective than unguided interventions. In China, ultrasound-guided acupotomy was more effective in reducing pain than unguided acupotomy for patients with knee osteoarthritis in several randomized controlled trials.^[8]^ In Korea, several retrospective studies reported positive effects of ultrasound-guided pharmacopuncture for the treatment of various musculoskeletal disorders, such as rib fracture and acute lower back pain.^[9–15]^

Through these reports, it can be inferred that ultrasound-guidance is actively applied in the clinical practice of KM. Fueled by the rapid development of science and technology, new technologies have been and adopted in medicine through multidisciplinary collaboration. In terms of interpreting statutes related to medical affairs in Korea, the emphasis is on an approach that reasonably expands the available options for users or consumers of healthcare services, rather than strictly dividing between Western and Korean medicine.^[16]^ In this context, there may emerge a new trend where doctors of Korean Medicine (KMDs) increasingly seek to provide treatments with enhanced safety and effectiveness by utilizing diagnostic medical imaging devices without depending on landmark palpation.

However, to date, no studies have reported on the current clinical use of ultrasound-guidance by KMDs. Currently in Korea, the use of ultrasound techniques by KMDs for medical purposes is not covered by the National Health Insurance (NHI). Thus, it is not possible to accurately identify the current clinical use of ultrasound-guided intervention by KMDs using data from the NHI database. Therefore, in this study, we conducted a survey on practicing KMDs who use ultrasound in their treatment, to examine the status of the use of ultrasound-guided intervention in KM, their experience of ultrasound-guided KM interventions, and opinions on NHI policies. In addition, the survey investigated diseases that benefited from the use of ultrasound-guidance in terms of effectiveness and safety of the intervention. Thus, the data and results of this study can be utilized as basic reference data for further clinical studies in the future.

## 2. Methods

### 2.1. Procedures

This study was based on an online survey conducted on KMDs practicing in various clinical settings of KM and utilizing ultrasound in their treatment. The survey questionnaire was developed through the researchers’ consensus and created in Moaform, an online survey platform. An email outlining the research purpose and including a survey link was sent to inform recipients about the study. In collaboration with the Association of Korean Medicine where all KMDs working in Korea are registered, the email was sent to all registered KMDs. Participants were allowed to proceed to the next page and start the survey only after they had read the study description and given their consent to participate. The expected number of survey respondents was 500, and our goal was to receive responses from over 300 KMDs who use ultrasound in their clinical practice. This study was approved by the Institutional Review Board of the Jaseng Hospital of Korean Medicine (Institutional Review Board file No. JASENG 2023-07-014).

### 2.2. Survey participants

#### 2.2.1. Inclusion criteria.

KMDs using ultrasound in their clinical practice and those who gave their consent to participate in this survey were included.

#### 2.2.2. Exclusion criteria.

KMDs not using ultrasound in their clinical practice, those who did not give their consent to participate in this survey, and those who deemed that survey participation was not appropriate, were excluded.

### 2.3. Survey items

The survey items were composed of questions in the following categories: Status of the adoption and utilization of ultrasound equipment, education and training, status of clinical use of ultrasound-guided intervention, safety of ultrasound-guided intervention, self-reported experience of the clinician, opinions on NHI reimbursement of ultrasound-guided intervention, and general characteristics of the respondents. More details of the survey items are presented in Table S1, Supplemental Digital Content, http://links.lww.com/MD/M48.

### 2.4. Statistical analysis

The Python package was used for statistical analysis for the survey. For each survey item, in the case of continuous variables, the means and standard deviations were calculated, and the median values were also presented considering the disparities in individual settings for clinical practice of KMDs. Frequencies and percentages were presented for the categorical variables.

## 3. Results

This survey was conducted from July 13, 2023 to August 16, 2023. In total, 609 KMDs responded to the email, and 606 KMDs gave their consent to participate. Out of these respondents, 374 (61.7%) stated that they currently use ultrasound devices in their clinical practice. Statistical analysis was performed with data of 335 KMDs, excluding 29 duplicate responses. Detailed results for the survey are presented in the supplementary materials.

### 3.1. Basic characteristics of participants

The respondents working in the capital area accounted for more than half (54.6%). Those working in KM clinics accounted for the highest proportion (N = 243, 72.5%), followed by KM hospitals (N = 76, 22.7%), public health institutions (N = 10, 3.0%), convalescent hospital (N = 4, 1.2%), and other types of medical institutions (N = 2, 0.6%). Clinic owners accounted for more than half of the respondents (N = 190, 56.7%), followed by employees (N = 118, 35.2%). Concerning the years of practice, the highest proportion of them worked 10 to 20 years (N = 123, 36.7%), followed by 5 to 10 (N = 93, 27.8%), ≤5 (N = 75, 22.4%), 20 to 30 (N = 30, 11.9%), and > 30 years (N = 4, 1.2%). Out of the respondents, 96 KMDs (28.7%) had a specialist certification (Table 1, Table S2, Supplemental Digital Content, http://links.lww.com/MD/M49).

**Table 1:** Basic characteristics of the included participants.

Variables	Responses	N	%
Area of practice	Capital area	183	54.6
	Cities	81	24.2
	Provinces	71	21.2
Type of medical institutions	Korean medicine clinics	243	72.5
	Korean medicine hospitals	76	22.7
	Public health institutions	10	3.0
	Convalescent hospital	4	1.2
	Others	2	0.6
Type of employment	Clinic owners	190	56.7
	Employees	122	36.4
	Public health doctors	9	2.7
	Residents	7	2.1
	Others	7	2.1
Years of practice	<5 yr	75	22.4
	5–10 yr	93	27.8
	10–20 yr	123	36.7
	20–30 yr	40	11.9
	≥30 yr	4	1.2
Specialist certification status	General practitioners	239	71.3
	Specialists	96	28.7

### 3.2. Status of the adoption and utilization of ultrasound equipment

**Figure 1.**
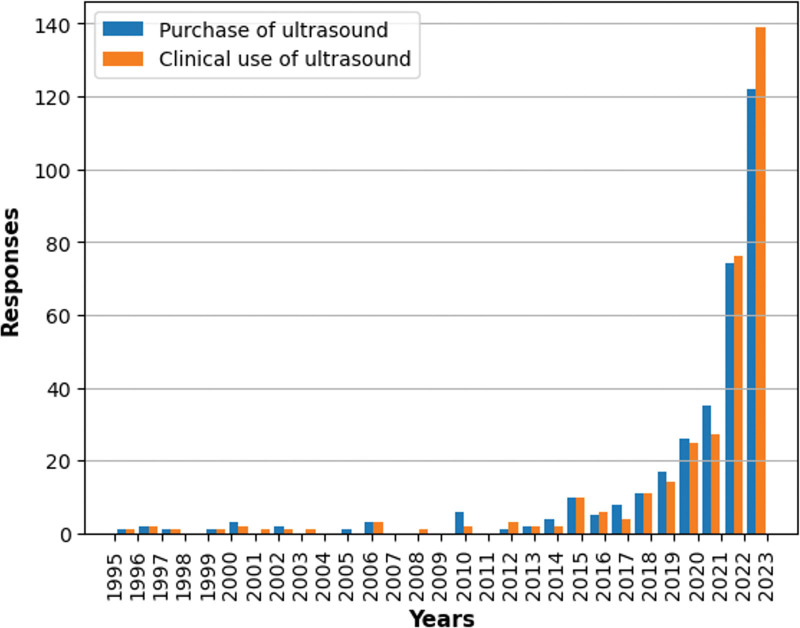
Time of ultrasound equipment purchase and clinical use of ultrasound.

From the responses, the earliest time to purchase the ultrasound equipment and its use in the clinical practice was in 1995. The use of ultrasound equipment began to increase slowly in the mid-2010s, and more than half of the respondents purchased and adopted the ultrasound equipment to their practice since 2022 (Fig. 1, Table S3, Supplemental Digital Content, http://links.lww.com/MD/M50). The average time taken from the purchase to its introduction for treatment was 0.3 ± 2 years. Currently, the mean number of patients treated with ultrasound-guided intervention was 14.5 ± 26.2 per week (median: 5).

### 3.3. Ultrasound-related education and training

The most frequently used sources of ultrasound training materials were books (N = 219, 65.4%) and private lectures for KMDs (N = 218, 65.1%). In addition, YouTube or other online lectures for health professionals (N = 142, 42.39%), study groups among KMDs (N = 99, 29.5%), continuing education programs for KMDs (N = 92, 27.5%), papers (N = 91, 27.2%), and conference (N = 68, 20.3%) were also used frequently. The mean time spent on training per week was 4.9 ± 4.5 (median: 3).

The total number of respondents with ultrasound-related certification was 95 (28.4%). The majority of certification holders had obtained the Registered in Musculoskeletal® sonography certification (92 respondents, 96.9%). This was followed by the Registry for Diagnostic Medical Sonography (RDMS) in Abdomen (17 respondents, 17.9%), RDMS in Obstetrics and Gynecology (3 respondents, 3.2%), RDMS in Breast (1 respondent, 1.1%), and Registered Vascular Technologist certifications (1 respondent, 1.1%). The average time taken to acquire an ultrasound-related certification was 11.0 ± 20.7 months, with a median of 6 months (Table S4, Supplemental Digital Content, http://links.lww.com/MD/M51).

### 3.4. Status of clinical use of ultrasound-guided KM intervention

The mean ratio of patients treated with ultrasound-guidance to the total number of patients visiting the KMD’s practice was 14.1 ± 16.8% (median 10%). For each patient, KMDs used ultrasound-guidance 4.7 ± 3.3 (median: 4) times in total for the entire treatment period, and 1.9 ± 1.1 (median: 2) times per week.

Respondents were asked to select their top 3 initial objectives for adopting ultrasound-guidance to KM interventions and to rate their choices. As a result, the primary objectives chosen by most KMDs (N = 183, 54.6%) were “To improve the accuracy and efficacy,” “To assess and diagnose” (N = 69, 20.6%), and “To use as an explanation tool for patients” (N = 49, 14.6%).

**Figure 2.**
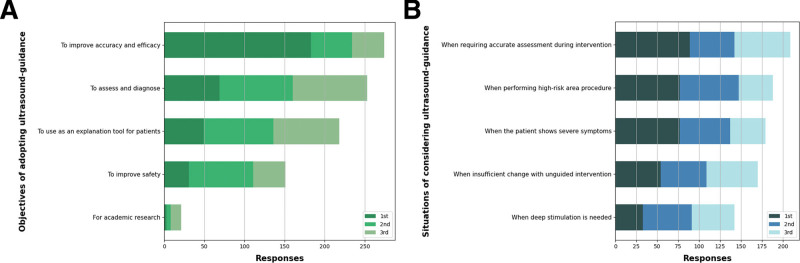
Reasons for using ultrasound-guidance in Korean medicine interventions. (A) The number of responses for the initial objectives of adopting ultrasound guidance to Korean medicine intervention; (B) The number of responses for situations of considering ultrasound-guided intervention in clinical practice.

Moreover, the respondents were asked to select and rate their top 3 situations for considering ultrasound-guided intervention in clinical practice. The most commonly chosen situation by KMDs (89 respondents, 26.6%) was “When requiring accurate assessment during intervention.” This was followed by 2 equally ranked situations: “When the patient shows severe symptoms,” and “When performing high-risk area procedure,” each selected by 77 respondents (23.0%) (Fig. 2, Table S5, Supplemental Digital Content, http://links.lww.com/MD/M52).

Regarding the top 3 body regions where ultrasound-guided intervention is most frequently used, the shoulder joint was selected by most KMDs (N = 168, 50.1%), followed by the knee joint (N = 84, 25.1%).

**Figure 3.**
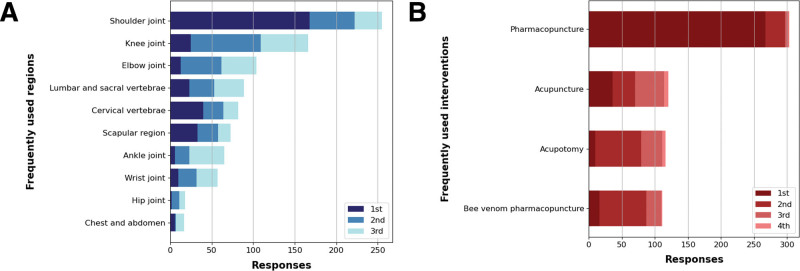
Usage patterns of ultrasound-guidance in Korean medicine interventions. (A) Shows the number of responses of the top 3 body regions where ultrasound-guided interventions are most frequently used. (B) Shows the top 3 types of Korean medicine interventions most frequently performed with ultrasound-guidance.

Concerning the top 3 KM interventions with frequent uses of ultrasound-guidance, pharmacopuncture was selected by most respondents (N = 267, 79.7%), followed by bee venom pharmacopuncture (N = 71, 21.2%) and acupotomy (N = 69, 20.6%), with the 2 interventions showing a similar proportion of selection (Fig. 3, Table S6, Supplemental Digital Content, http://links.lww.com/MD/M53).

Additionally, to determine future clinical research themes, KMDs were asked through an open-ended question to specify diseases and body regions where the clinical effects of ultrasound-guided KM interventions were significantly improved compared to unguided KM interventions. The answers provided by the KMDs were organized by disease and region of the body, and those without a clear indication of the disease or region were excluded from the counts. As for the disease showing a significant difference in clinical effects with ultrasound-guidance, nerve entrapment (region unspecified) was selected by most KMDs (N = 38), followed by subacromial-subdeltoid bursitis (N = 27), frozen shoulder (adhesive capsulitis) (N = 24), tennis elbow (N = 20), and rotator cuff tear (N = 15). When the answers were classified by the region in the body, the shoulder joint was selected by most KMDs (N = 157), followed by the elbow joint (N = 31) and lumbar vertebrae (N = 27) (Table 2).

**Table 2:** Diseases and regions in the body show a significant improvement in clinical effects when using ultrasound-guidance for Korean medicine interventions compared to the effects from unguided Korean medicine interventions.

Rank	Disease		Rank	Region	
	Disease	Case		Region	Case
1	Nerve entrapment (region unspecified)	38	1	Shoulder joint	157
2	Subacromial-subdeltoid bursitis	27	2	Elbow joint	31
3	Frozen shoulder	24	3	Lumbar vertebrae	27
4	Lateral epicondylitis	20	4	Hand	12
5	Rotator cuff tear	15	5	Wrist joint	12
6	Lumbar disc herniation, radiculopathy	12	6	Knee joint	11
7	Carpal tunnel syndrome	11	7	Ankle joint	6
8	Rotator cuff tendinitis	10	8	Cervical vertebrae	5
9	Impingement syndrome	9			
10	Trigger finger	8			

In addition, among KMDs using ultrasound equipment in their treatment, the cases of using ultrasound for the purpose limited to guidance accounted for 29.5 ± 29.5% (median: 20%). Further, the survey inquired about the types of ultrasound examinations KMDs employed for diagnostic purposes, as opposed to guidance. The most frequently used examinations were musculoskeletal ultrasound (N = 309, 92.2%), abdominal ultrasound (N = 167, 49.9%), and female pelvic ultrasound (N = 60, 17.9%) (Table S7, http://links.lww.com/MD/M54).

### 3.5. Safety of ultrasound-guided intervention

In the survey, KMDs were also asked about their experience of adverse events (AEs) during ultrasound-guided interventions. Regarding the frequency of experiencing AEs with sequelae, most respondents indicated “Rarely” (N = 319, 95.2%), followed by “Occasionally” (N = 14, 4.2%) and “Sometimes” (N = 2, 0.6%). Concerning the types of AEs, the most commonly reported were “pain at the area of treatment (e.g. needle sites)” (N = 246, 73.4%) and “bruise or hematoma at the area of treatment” (N = 209, 62.4%; Table 3).

**Table 3:** Adverse events from ultrasound-guided intervention.

Frequency of long-term adverse events					
	Rarely	Occasionally	Sometimes	Frequently	Nearly always
N	319	14	2	0	0
%	95.2	4.2	0.6	0.0	0.0

Types of adverse events				N	%
Pain at the area of treatment				264	73.4
Bruise or hematoma at the area of treatment				209	62.4
Infection at the area of treatment (swelling, heat sensation)				34	10.1
Paresthesia				49	14.6
Worsening of existing symptoms				38	11.3
Tiredness/drowsiness				29	8.7

### 3.6. Self-reported experience of the clinician for ultrasound-guided intervention

In the survey, we also asked regarding the subjective experience of the KMDs for using ultrasound-guidance in KM interventions. Specifically, we focused on aspects of safety, effectiveness, expertise, and patient satisfaction compared to unguided interventions.

In terms of safety, 94.0% of KMDs responded positively, comprising “much improved” (N = 143, 42.7%), “very much improved” (N = 107, 31.9%), and “slightly improved” (N = 65, 19.4%).

In terms of effectiveness, 94.0% of KMDs responded positively, comprising “much improved” (N = 115, 34.3%), “slightly improved” (N = 115, 30.5%), and “very much improved” (N = 98, 29.3%).

In terms of expertise as a health professional, 97.3% of KMDs responded positively, comprising “very much improved” (N = 163, 48.7%), “much improved” (N = 125, 37.3%), and “slightly improved” (N = 37, 11.3%).

**Figure 4.**
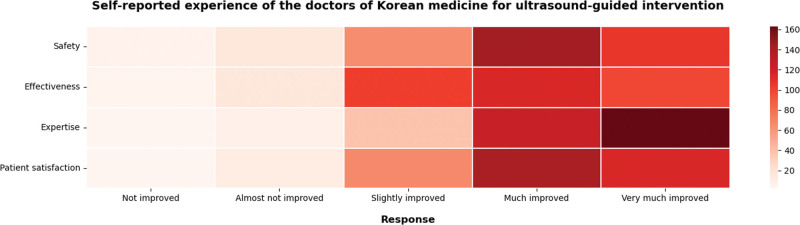
Self-reported experience of doctors of Korean medicine for ultrasound-guided intervention. The figure shows the response trends rating the degree of change in safety, effectiveness, expertise, and patient satisfaction after introducing ultrasound-guidance to Korean medicine interventions.

Concerning patient satisfaction, 96.1% of KMDs responded positively, comprising “much improved” (N = 140, 41.8%), “very much improved” (N = 115, 34.3%), and “slightly improved” (N = 67, 20.0%) (Fig. 4, Table S8, Supplemental Digital Content, http://links.lww.com/MD/M55).

For a basic assessment of the relative work value involved in using ultrasound-guidance, we asked the KMDs how much more time and efforts (including practice intensity, mental and physical stress, and clinical judgment requirements) were needed when using ultrasound-guidance compared to unguided interventions. The answers showed that 3.3 ± 2.2 (median: 3) and 4.0 ± 5.9 (median: 3) times more time and efforts, respectively, than unguided interventions.

### 3.7. Opinions on NHI reimbursement of ultrasound-guided intervention and the fee for service

Regarding the necessity of NHI reimbursement of ultrasound-guided KM intervention as a medical service, 94.9% of KMDs responded positively. This consisted of “Strongly Agree” (N = 280, 83.6%) and “Agree” (N = 38, 11.3 %) (Table S9, Supplemental Digital Content, http://links.lww.com/MD/M56). The reasons for disagreement included concerns about an “increased burden of patients” and “failing to have adequate pricing for the fee of the ultrasound-guided KM intervention.”

Based on the cases of treatment for shoulder joints, the survey also asked for an appropriate fee for ultrasound-guided acupuncture, ultrasound-guided pharmacopuncture, and ultrasound-guided acupotomy. For ultrasound-guided acupuncture, ultrasound-guided pharmacopuncture, and ultrasound-guided acupotomy, the respondents indicated fee ranges as follows: “USD 7.66 to 22.97 (KRW 10,000 to 30,000)” for acupuncture (N = 113, 39.7%), “USD 22.97 to 38.28 (KRW 30,000 to 50,000)” for pharmacopuncture (N = 105, 31.3%), and “USD 22.97 to 38.28 (KRW 30,000 to 50,000)” (N = 83, 24.8%) with “More than USD 76.57 (KRW ≥ 100,000)” (N = 77, 23.0%) being the most common response for acupotomy (Table S9, Supplemental Digital Content, http://links.lww.com/MD/M56, Figure S1, Supplemental Digital Content, http://links.lww.com/MD/M57).

## 4. Discussion

Ultrasound-guided KM intervention represents a significant development in traditional KM, showcasing the integration of modern science and technology. It serves as a symbolic example of the paradigm shift in traditional acupuncture technique, which historically relied mainly on palpation for assessment and diagnosis. In this context, this study makes a notable contribution to the literature as the first to investigate the clinical practice of ultrasound-guided KM intervention among KMDs.

An introductory email on the survey was sent to all KMDs members of the Association of Korean Medicine, and 609 KMDs responded over 1 month. As of 2020, the number of KMDs practicing in medical institutions is 20,101,^[17]^ and considering the annual increase of 626 KMDs on an average, the number of KMDs practicing in medical institutions in 2023 is expected to be around 21,353. Based on this estimation, the survey response rate is approximately 2.9%. The majority of respondents were working in KM clinics (72.5%) and KM hospitals (22.7%).

Initially, to specifically target KMDs who use ultrasound in their practice, we included a question about their use of ultrasound equipment. Among the survey participants, 40.9% reported using ultrasound in their practice. Prior to survey initiation, we ensured that the survey focused only on KMDs who were using ultrasound in their treatment, and anticipating a lower participation rate from those uninterested in ultrasound usage. We should note that the 40.9% response rate does not represent the actual rate of KMDs using ultrasound in clinical practice out of the entire population of KMDs.

As there had been no previous investigation into KMDs’ clinical use of ultrasound equipment, we first tried to find out the time when ultrasound equipment was purchased and introduced into KM practice. From 1995 to 2015, few respondents introduced ultrasound per year; however, from 2016, there was a marked trend of a gradual increase and more than half of the respondents (58.5%) introduced ultrasound into their practice in 2022 and 2023 This trend aligns with the emergence of research on standardizing ultrasound use for critical acupuncture points, reported in Korea in 2017,^[7]^ and a surge in the publication of case series/reports utilizing ultrasound-guidance from 2019.^[2,3,11,13]^ The progression in ultrasound usage in KM practice can be summarized as follows: initial research focused on standardization, followed by case studies on ultrasound application in KM interventions. The trend significantly accelerated after the Supreme Court’s ruling that KMDs’ use of ultrasonic devices does not violate the Medical Service Act,^[16]^ leading to more active adoption in KM clinical practice.

Respondents indicated treating an average of 14.5 ± 26.2 (median: 5) patients per week with ultrasound-guided procedures, and the number ranged widely from less than 1 to 280. Through these answers, we can see that there was a remarkable difference in the utilization status of ultrasound among the respondents. These variability might have been caused by the proficiency, primarily practicing fields, or specializations.

For ultrasound training, most KMDs relied on books (65.4%) and private lectures (65.1%). About 28.4% of the respondents acquired ultrasound-related certification and most (96.9%) of them received Registered in Musculoskeletal, reflecting the high prevalence of musculoskeletal disorders in KM outpatient care.^[18]^ It can be inferred that KMDs acquired certification according to their clinical practice field and by attending private lectures for KMDs, they tried to gain knowledge and techniques that can be immediately utilized. KMDs spent 4.9 ± 4.5 hours per week on average for training. Considering that many of the KMDs adopted ultrasound in their practice in the last 1 or 2 years, they might tend to spend a considerable amount of time in training.

More than half of the KMDs (54.6%) cited “improving accuracy and efficacy of the procedure” as their primary objective for adopting ultrasound-guidance, showing that clinical effectiveness was the most significant factor when ultrasound was first introduced. At the same time, ultrasound may have enabled treatment in high-risk areas previously inaccessible with blind procedures, while ensuring safety. Meanwhile, the most common situations for considering ultrasound-guidance in practice were “When accurate assessment and diagnosis of the patient is required at the same time as administering the intervention” (26.6%), “When performing procedures in high-risk regions” and “When the patient shows severe symptoms,” (each 23.0%). Thus, in real-world practice, the KMDs consider ultrasound-guidance in a range of situations depending on the individual conditions of the patients and the circumstances of administering the intervention.

The shoulder joint was the most frequently targeted area by ultrasound-guidance, selected by 50.1% of the KMDs. Further, the shoulder joint was selected by most KMDs as the region showing a significant difference in clinical effectiveness between ultrasound-guided and unguided treatment. Owing to the complex anatomical structure of the shoulder joints, researches on the use of ultrasound-guidance injections have been conducted in western medicine. Previous studies have also reported that ultrasound-guidance injections demonstrated significantly higher accuracy and efficacy compared to those of blind for the treatment of shoulder joint diseases.^[19,20]^ The number of total responses was in the order of shoulder joint, knee joint, elbow joint, lumbar and sacral vertebrae, and cervical vertebrae, and the joints with complex anatomical structures and lumbar vertebrae, a high-risk region, were in the top choices of the regions selected by the KMDs.

Pharmacopuncture emerged as the most commonly chosen KM intervention using ultrasound-guidance (79.9%). Pharmacopuncture often targets specific lesion, including Ashi points for treatment during the procedure, and the significance of accurate localization, fixture, and penetration of the targeted tissue has been emphasized.^[21]^ Considering that the primary objective of introducing ultrasound was “To improve the accuracy and efficacy of the procedure,” the response from the KMDs confirmed that ultrasound has been used to improve the quality of pharmacopuncture procedure in the joints with complex anatomical structures or in high-risk regions.

An open-ended question format was used to find out diseases showing greater clinical effectiveness with the use of ultrasound-guidance in KM interventions than that with unguided interventions. After examining the top 5 choices, there were 38 answers that recorded “nerve entrapment syndrome” without specifying the region, and three out of the top 5 choices were shoulder joint diseases (subacromial-subdeltoid bursitis ranked second, frozen shoulder ranked third, and rotator cuff tear ranked fifth). Lateral epicondylitis ranked fourth in the responses provided by the KMDs.

There have been reports in several studies that pharmacopuncture is effective for treating nerve entrapment syndrome.^[22–25]^ In the field of medicine, it is also one of the cases where the safety and effectiveness of commonly used injectates have been enhanced through ultrasound-guidance.^[26]^ In the case of shoulder joint disorders, in KM, there have been reports of a significant improvement in pain and range of motion by administering ultrasound-guided soyeom pharmacopuncture at the acromioclavicular joint for 9 patients with anterior shoulder pain.^[9]^ As aforementioned, ultrasound-guidance has been actively utilized for the treatment of shoulder pathology in medical fields.^[19,20]^ It has also been effectively used in the treatment of lateral epicondylitis, acupuncture,^[27]^ and pharmacopuncture.^[28]^ There was also a previous study reporting that acupotomy showed a lower rate of recurrence than that in corticosteroid injections for the treatment of tennis elbow.^[29]^ In the same context, ultrasound-guidance has been actively utilized to enable the administration of minimally invasive percutaneous needle puncture when injecting for the treatment of lateral epicondylitis.^[30,31]^

As above, the diseases that KMDs selected as those showing a significant improvement in the effectiveness of KM interventions with the application of ultrasound-guidance were the same as those which has been reported enhanced effectiveness and safety through ultrasound-guidance in medical fields. Thus, it is expected that in the future, if the findings from medical studies, such as ultrasound-guidance injection or hydro-dissection, can be applied or utilized in KM, the effectiveness and safety of KM interventions will also be enhanced, resulting in practical benefits for the patients.

Safety-wise, 95.2% of KMDs reported almost no AEs with sequelae by ultrasound-guided interventions. In formulating the multiple-choice question on AEs, the authors referred to previous studies,^[32–35]^ and provided options including pain at the treatment area, bruise, hematoma, swelling, heat sensation, paresthesia, worsening of existing symptoms, tiredness/drowsiness, among others. Our findings revealed that the most commonly reported AEs by KMDs were pain at the treatment site (73.4%) and bruising or hematoma (62.4%). As improvement of safety is one of the main purpose for using ultrasound-guidance, future surveys should explore the differences in the occurrence of specific AEs between ultrasound-guided KM interventions and unguided interventions.

KMDs reported improvements of KM treatments in terms of safety, effectiveness, expertise, and patient satisfaction by introducing ultrasound-guidance. Further, in a survey conducted in Korea that investigated public perceptions on the use of diagnostic devices by KMDs in 2002, 83.3% responded that there is a need for authorizing KMDs to use diagnostic ultrasound.^[36]^ Consequently, the use of ultrasound by KMDs not only has a positive effect on the clinical effectiveness and safety of KM interventions, but also contributes to meeting the needs of patients.

When asked about the required time and effort, KMDs reported that the introduction of ultrasound-guidance increased the time by 3.3 ± 2.2 times and the effort by 4.0 ± 5.9 times compared to unguided interventions. These responses confirm that ultrasound-guided KM intervention demands significantly more time and effort than unguided approaches. Therefore, 94.9% of the respondents expressed their agreement on NHI reimbursement for the fee of the ultrasound-guided KM interventions. The KMDs responded that the fee should be priced in the order of acupotomy, pharmacopuncture, and general acupuncture, reflecting the complexity and difficulty of each procedure. To accurately price these interventions, a more rigorous valuation is required, considering factors such as the difficulty of the intervention, the region and diseases, as well as the number of treatment points.

Additionally, the survey included a brief question about the use of ultrasound for diagnostic purposes beyond guidance. It was most frequently used for diagnosing musculoskeletal system disorders (92.2%), which mirrored its usage in guidance. Abdominal ultrasound was used by 49.9% of KMDs, indicating its application in internal medicine treatments. This suggests the need for a more comprehensive survey to explore ultrasound use in diagnosing diseases beyond musculoskeletal disorders.

This study had several limitations. Firstly, as this study was the first survey research with an online survey to investigate the overall perceptions of KMDs on the use of ultrasound, we simplified the questions to facilitate understanding. For example, the questions included the frequency of ultrasound-guided interventions and the total number of interventions for each patient, but this approach may have resulted in a wide range of responses due to varying diseases and patient conditions. Thus, future surveys should include disease-specific questions to obtain more precise data. Additionally, the responses might have been influenced by the KMDs’ main practice areas, suggesting a need for focused interviews with KMDs having extensive clinical experience in using ultrasound for specific diseases. Secondly, the survey questions rely heavily on memory and subjective opinions, potentially leading to recall and self-report biases. Thirdly, with a low response rate of 2.9% for this survey, there’s a potential for nonresponse bias. KMDs less frequent in using email might have had fewer opportunities to participate. Therefore, future studies using prospective clinical trials or real-world data might yield different results. Finally, KMDs currently in training but not applying ultrasound in their clinical practice were not included. Including these individuals in future research could provide valuable insights into the evolving trends and future directions of ultrasound-guided KM interventions.

In Korea, traditional KM services are mainly provided on the primary care level. Thus, although KMDs may appreciate the usefulness of ultrasound-guided KM intervention in their clinical practice, in reality, it is difficult to build relevant evidence based on well-designed randomized controlled trials in a short time. Nevertheless, our findings confirmed that ultrasound-guidance is actively applied in KM clinics, which points to the necessity of establishing a practice-based research network. As revealed in the survey responses of this study, ultrasound-guided KM interventions have begun to be widely adopted within the last 1 to 2 years. Under these circumstances, this study is the first large-scale survey to investigate the status of ultrasound-guided KM interventions and the current clinical use of ultrasound equipment by KMDs. This study will serve as instrumental basic reference data to derive practice based-clinical questions for further clinical studies and developing clinical practice guidelines.

## Acknowledgments

We thank all the doctors of Korean medicine who sincerely shared their clinical experiences to contribute to the advancement of Korean medicine.

## Author contributions

**Conceptualization:** Ju Yeon Kim, Jung Min Yun, In Heo, Woo-Chul Shin, Jae-Heung Cho, Byung-Kwan Seo, In-Hyuk Ha.

**Data curation:** Jung Min Yun.

**Formal analysis:** Ju Yeon Kim.

**Funding acquisition:** In-Hyuk Ha.

**Investigation:** Ju Yeon Kim.

**Methodology:** Ju Yeon Kim, Sook-Hyun Lee, Dong Kun Ko.

**Project administration:** Ju Yeon Kim.

**Resources:** Jung Min Yun, Sook-Hyun Lee, Yoon Jae Lee.

**Software:** Ju Yeon Kim, Jung Min Yun, Sook-Hyun Lee.

**Supervision:** Yoon Jae Lee, In-Hyuk Ha.

**Validation:** Jung Min Yun, Yoon Jae Lee.

**Visualization:** Ju Yeon Kim.

**Writing – original draft:** Ju Yeon Kim.

**Writing – review & editing:** Ju Yeon Kim, Dong Kun Ko, In Heo, Woo-Chul Shin, Jae-Heung Cho, Byung-Kwan Seo, In-Hyuk Ha.

## Supplementary Material



















**Figure:**