# Supplementary material for: Survey on the current usage of ultrasound-guided procedures in Korean Medicine Clinics and Hospitals
Source: Medicine (Baltimore). 2024 Apr 5;103(14):e37659. doi: 10.1097/MD.0000000000037659 (PMC10994457; doi:10.1097/MD.0000000000037659)
Supplement: Supplementary file 1 [file medi-103-e37659-s001.docx]

**Supplementary Table 1.** Survey items

| **Category** | **Questions** |
| --- | --- |
| 1. Adoption and utilization of ultrasound equipment | (1) Usage of ultrasound equipment in clinical practice  (2) Time of purchasing the ultrasound equipment and introduction to clinical practice  (3) Number of patients treated with ultrasound-guided intervention per week |
| 2. Education and training | (1) Source of education and training materials on ultrasound  (2) Time spent on ultrasound training per week  (3) Status of ultrasound-guidance-related certification, type of certification, time of acquisition |
| 3. Clinical use of ultrasound-guided intervention | (1) Ratio of patients treated with ultrasound-guidance to the total number of patients  (2) Total and frequency of using ultrasound-guidance per patient  (3) Objectives, clinical situations for considering use of ultrasound-guidance, frequent uses  (4) Disease with significant improvement with ultrasound-guidance  (5) Frequently used ultrasound examination procedures for diagnostic purposes |
| 4. Safety of ultrasound-guided intervention | (1) Frequency of adverse events with sequelae (AEs)  (2) Frequently reported AEs |
| 5. Self-reported experience of clinicians using ultrasound-guided intervention | (1) Experience with safety, effectiveness, expertise, and patient satisfaction  (2) Time and effort spent for the use of ultrasound-guidance |
| 6. Opinions on NHI reimbursement of ultrasound-guided intervention and the fee for service | (1) Degree of agreement on the necessity for NHI reimbursement  (2) Opinions on the adequate pricing of the fee for ultrasound-guided KM intervention |
| 7. General characteristics of the respondents | (1) Sex  (2) Age group  (3) Area of practice  (4) Type of medical institutions  (5) Type of employment  (6) Years of practice  (7) Specialist certification status |

**AE, Adverse event; NHI, National health insurance; KM, Korean medicine**
