# Supplementary material for: Survey on the current usage of ultrasound-guided procedures in Korean Medicine Clinics and Hospitals
Source: Medicine (Baltimore). 2024 Apr 5;103(14):e37659. doi: 10.1097/MD.0000000000037659 (PMC10994457; doi:10.1097/MD.0000000000037659)
Supplement: Supplementary file 2 [file medi-103-e37659-s002.docx]

**Supplementary Table 2.** Basic characteristics of the included participants

| Variables | Responses | N | % |
| --- | --- | --- | --- |
| Sex | Male | 275 | 82.1 |
|  | Female | 60 | 17.9 |
| Age group | 20–29 | 24 | 7.2 |
|  | 30–39 | 152 | 45.4 |
|  | 40–49 | 124 | 37.0 |
|  | 50–59 | 28 | 8.4 |
|  | 60–69 | 7 | 2.1 |
|  | ≥70 years | 0 | 0 |
| Area of practice | Seoul | 99 | 29.6 |
|  | Busan | 14 | 4.2 |
|  | Daegu | 20 | 6.0 |
|  | Incheon | 19 | 5.7 |
|  | Gwangju | 6 | 1.8 |
|  | Daejeon | 13 | 3.9 |
|  | Ulsan | 8 | 2.4 |
|  | Gyeonggi | 84 | 25.1 |
|  | Gangwon | 6 | 1.8 |
|  | Chungbuk | 8 | 2.4 |
|  | Chungnam | 13 | 3.9 |
|  | Jeonbuk | 8 | 2.4 |
|  | Jeonnam | 11 | 3.3 |
|  | Gyeongbuk | 15 | 4.5 |
|  | Gyeongnam | 9 | 2.7 |
|  | Jeju | 1 | 0.3 |
|  | Sejong | 1 | 0.3 |
| Type of medical institutions | Korean Medicine clinics | 243 | 72.5 |
|  | Korean Medicine hospitals | 76 | 22.7 |
|  | Public health institutions | 10 | 3.0 |
|  | Convalescent hospital | 4 | 1.2 |
|  | Others | 2 | 0.6 |
| Type of employment | Clinic owners | 190 | 56.7 |
|  | Employees | 122 | 36.4 |
|  | Public health doctors | 9 | 2.7 |
|  | Residents | 7 | 2.1 |
|  | Others | 7 | 2.1 |
| Years of practice | <5 years | 75 | 22.4 |
|  | 5–10 years | 93 | 27.8 |
|  | 10–20 years | 123 | 36.7 |
|  | 20–30 years | 40 | 11.9 |
|  | ≥30 years | 4 | 1.2 |
| Specialist certification status | General practitioners | 239 | 71.3 |
|  | Specialists | 96 | 28.7 |
| Specialty | Korean Medicine Rehabilitation | 22 | 22.9 |
|  | Acupuncture and Moxibustion | 28 | 29.2 |
|  | Internal Korean Medicine | 21 | 21.9 |
|  | Korean Medicine Gynecology | 8 | 8.3 |
|  | Korean Medicine Pediatrics | 2 | 2.1 |
|  | Korean Medicine Neuropsychiatry | 4 | 4.2 |
|  | Korean Medicine Ophthalmology & Otorhinolaryngology and Dermatology | 3 | 3.1 |
|  | Sasang Constitutional Medicine | 8 | 8.3 |
