# Supplementary material for: Survey on the current usage of ultrasound-guided procedures in Korean Medicine Clinics and Hospitals
Source: Medicine (Baltimore). 2024 Apr 5;103(14):e37659. doi: 10.1097/MD.0000000000037659 (PMC10994457; doi:10.1097/MD.0000000000037659)
Supplement: Supplementary file 3 [file medi-103-e37659-s003.docx]

**Supplementary Table 3.** Time of ultrasound equipment purchase and clinical use of ultrasound

| Year | Purchase of ultrasound equipment | | Clinical use of ultrasound equipment | |
| --- | --- | --- | --- | --- |
|  | N | % | N | % |
| 1995 | 1 | 0.3 | 1 | 0.3 |
| 1996 | 2 | 0.6 | 2 | 0.6 |
| 1997 | 1 | 0.3 | 1 | 0.3 |
| 1998 | 0 | 0 | 0 | 0 |
| 1999 | 1 | 0.3 | 1 | 0.3 |
| 2000 | 3 | 0.9 | 2 | 0.6 |
| 2001 | 0 | 0 | 1 | 0.3 |
| 2002 | 2 | 0.6 | 1 | 0.3 |
| 2003 | 0 | 0 | 1 | 0.3 |
| 2004 | 0 | 0 | 0 | 0 |
| 2005 | 1 | 0.3 | 0 | 0 |
| 2006 | 3 | 0.9 | 3 | 0.9 |
| 2007 | 0 | 0 | 0 | 0 |
| 2008 | 0 | 0 | 1 | 0.3 |
| 2009 | 0 | 0 | 0 | 0 |
| 2010 | 6 | 1.8 | 2 | 0.6 |
| 2011 | 0 | 0 | 0 | 0 |
| 2012 | 1 | 0.3 | 3 | 0.9 |
| 2013 | 2 | 0.6 | 2 | 0.6 |
| 2014 | 4 | 1.2 | 2 | 0.6 |
| 2015 | 10 | 3.0 | 10 | 3.0 |
| 2016 | 5 | 1.5 | 6 | 1.8 |
| 2017 | 8 | 2.4 | 4 | 1.2 |
| 2018 | 11 | 3.3 | 11 | 3.3 |
| 2019 | 17 | 5.1 | 14 | 4.2 |
| 2020 | 26 | 7.8 | 25 | 7.5 |
| 2021 | 35 | 10.4 | 27 | 8.1 |
| 2022 | 74 | 22.1 | 76 | 22.7 |
| 2023 | 122 | 36.4 | 139 | 41.5 |
