# Supplementary material for: Survey on the current usage of ultrasound-guided procedures in Korean Medicine Clinics and Hospitals
Source: Medicine (Baltimore). 2024 Apr 5;103(14):e37659. doi: 10.1097/MD.0000000000037659 (PMC10994457; doi:10.1097/MD.0000000000037659)
Supplement: Supplementary file 4 [file medi-103-e37659-s004.docx]

**Supplementary Table 4.** Education/training of KMDs related to ultrasound

| Variables | Responses | N | % |
| --- | --- | --- | --- |
| Source of ultrasound training materials | Books | 219 | 65.4 |
|  | Private lectures for KMDs | 218 | 65.1 |
|  | YouTube or other online lectures for health professionals | 142 | 42.4 |
|  | Study groups among KMDs | 99 | 29.6 |
|  | Continuing education programs for KMDs | 92 | 27.5 |
|  | Papers (academic publications) | 91 | 27.2 |
|  | Conference training materials | 68 | 20.3 |
| Time spent on ultrasound training per week | 4.9±4.5 h on average (median 3) | | |
| Status of ultrasound guidance-related certification | Yes | 95 | 28.4 |
|  | No | 240 | 71.6 |
| Number of certifications | 1 | 79 | 83.2 |
|  | 2 | 13 | 13.7 |
|  | 3 | 3 | 3.2 |
| Type of certification | RMSK | 92 | 96.8 |
|  | RDMS (Abdomen) | 17 | 17.9 |
|  | RDMS (Obstetrics and gynecology) | 3 | 3.2 |
|  | RDMS (Breast) | 1 | 1.0 |
|  | RVT | 1 | 1.0 |
| Time taken to acquire the certification | 11.0±20.7 months on average (median: 6) |  |  |

KMDs, Doctors of Korean medicine; RMDS, Registered Diagnostic Medical Sonographer, RMSK, Registered in Musculoskeletal sonography; RVT, Registered Vascular Technologist
