# Supplementary material for: Survey on the current usage of ultrasound-guided procedures in Korean Medicine Clinics and Hospitals
Source: Medicine (Baltimore). 2024 Apr 5;103(14):e37659. doi: 10.1097/MD.0000000000037659 (PMC10994457; doi:10.1097/MD.0000000000037659)
Supplement: Supplementary file 5 [file medi-103-e37659-s005.docx]

**Supplementary Table 5.** Reasons for using ultrasound-guidance in Korean medicine interventions

| Variables | Total | | 1st | | 2nd | | 3rd | |
| --- | --- | --- | --- | --- | --- | --- | --- | --- |
|  | **N** | **%** | **N** | **%** | **N** | **%** | **N** | **%** |
| Initial objectives of adopting ultrasound guidance to Korean medicine interventions | | | | | | | | |
| To improve the accuracy and efficacy | 274 | 81.8 | 183 | 54.6 | 51 | 15.2 | 40 | 11.9 |
| To assess and diagnose | 253 | 75.5 | 69 | 20.6 | 91 | 27.2 | 93 | 27.8 |
| To use as an explanation tool for patients | 218 | 65.1 | 49 | 14.6 | 87 | 26.0 | 82 | 24.5 |
| To improve safety | 151 | 54.1 | 31 | 9.3 | 80 | 23.9 | 40 | 11.9 |
| For academic research | 21 | 6.3 | 3 | 0.9 | 5 | 1.5 | 13 | 3.9 |
| Situations of considering ultrasound-guided intervention in clinical practice | | | | | | | | |
| When requiring accurate assessment during intervention | 209 | 62.4 | 89 | 26.6 | 53 | 15.8 | 67 | 20.0 |
| When performing high-risk area procedure | 188 | 56.1 | 77 | 23.0 | 70 | 20.9 | 41 | 12.2 |
| When the patient shows severe symptoms | 179 | 53.4 | 77 | 23.0 | 60 | 17.9 | 42 | 12.5 |
| When insufficient change with unguided intervention | 170 | 50.7 | 54 | 16.1 | 55 | 16.4 | 61 | 18.2 |
| When deep stimulation is needed | 142 | 42.4 | 33 | 9.9 | 58 | 17.3 | 51 | 15.2 |
