# Supplementary material for: Survey on the current usage of ultrasound-guided procedures in Korean Medicine Clinics and Hospitals
Source: Medicine (Baltimore). 2024 Apr 5;103(14):e37659. doi: 10.1097/MD.0000000000037659 (PMC10994457; doi:10.1097/MD.0000000000037659)
Supplement: Supplementary file 6 [file medi-103-e37659-s006.docx]

**Supplementary Table 6.** Usage patterns of ultrasound-guidance in Korean medicine interventions

| Regions in the body with frequent uses of ultrasound guidance | | | | | | | | | | | | | | | | | |
| --- | --- | --- | --- | --- | --- | --- | --- | --- | --- | --- | --- | --- | --- | --- | --- | --- | --- |
| Variables | | **Total** | | | | **1st** | | | | **2nd** | | | | **3rd** | | | |
|  |  | **N** | | **%** | | **N** | | **%** | | **N** | | **%** | | **N** | | **%** | |
|  | Shoulder joint | 255 | | 76.1 | | 168 | | 50.1 | | 54 | | 16.1 | | 33 | | 9.9 | |
|  | Knee joint | 166 | | 49.6 | | 25 | | 7.5 | | 84 | | 25.1 | | 57 | | 17.0 | |
|  | Elbow joint | 104 | | 31.0 | | 13 | | 3.9 | | 49 | | 14.6 | | 42 | | 12.5 | |
|  | Lumbar and sacral vertebrae | 89 | | 26.6 | | 23 | | 6.9 | | 30 | | 9.0 | | 36 | | 10.7 | |
|  | Cervical vertebrae | 82 | | 24.5 | | 40 | | 11.9 | | 24 | | 7.2 | | 18 | | 5.4 | |
|  | Scapula region | 73 | | 21.8 | | 33 | | 9.9 | | 25 | | 7.5 | | 15 | | 4.5 | |
|  | Ankle joint | 65 | | 19.4 | | 6 | | 1.8 | | 17 | | 5.1 | | 42 | | 12.5 | |
|  | Wrist joint | 57 | | 17.0 | | 10 | | 3.0 | | 22 | | 6.6 | | 25 | | 7.5 | |
|  | Hip joint | 18 | | 5.4 | | 2 | | 0.6 | | 9 | | 2.7 | | 7 | | 2.1 | |
|  | Chest and abdomen | 17 | | 5.1 | | 6 | | 1.8 | | 1 | | 0.2 | | 10 | | 3.0 | |
| Types of Korean medicine interventions with frequent uses of ultrasound guidance | | | | | | | | | | | | | | | | | |
| Variables | | **Total** | | | **1st** | | | | **2nd** | | **3rd** | | | | **4th** | | |
|  |  | **N** | **%** | | **N** | | **%** | | **N** | **%** | **N** | | **%** | | **N** | | **%** |
|  | Pharmacopuncture | 303 | 90.4 | | 267 | | 79.7 | | 30 | 9.0 | 5 | | 1.5 | | 1 | | 0.3 |
|  | Acupuncture | 120 | 35.8 | | 36 | | 10.7 | | 34 | 10.1 | 44 | | 13.1 | | 6 | | 1.8 |
|  | Acupotomy | 116 | 34.6 | | 10 | | 3.0 | | 69 | 20.6 | 32 | | 9.6 | | 5 | | 1.5 |
|  | Bee venom pharmacopuncture | 111 | 33.1 | | 16 | | 4.8 | | 71 | 21.2 | 23 | | 6.9 | | 1 | | 0.3 |
