# Supplementary material for: Survey on the current usage of ultrasound-guided procedures in Korean Medicine Clinics and Hospitals
Source: Medicine (Baltimore). 2024 Apr 5;103(14):e37659. doi: 10.1097/MD.0000000000037659 (PMC10994457; doi:10.1097/MD.0000000000037659)
Supplement: Supplementary file 7 [file medi-103-e37659-s007.docx]

**Supplementary Table 7.** Types of frequently used ultrasound examination

| Exam type | Total | | 1st | | 2nd | | 3rd | |
| --- | --- | --- | --- | --- | --- | --- | --- | --- |
|  | **N** | **%** | **N** | **%** | **N** | **%** | **N** | **%** |
| Musculoskeletal | 309 | 92.2 | 266 | 79.4 | 32 | 9.6 | 11 | 3.3 |
| Abdominal | 167 | 49.9 | 55 | 16.4 | 89 | 26.6 | 23 | 6.9 |
| Female pelvic | 60 | 17.9 | 8 | 2.4 | 17 | 5.1 | 35 | 10.4 |
| Thyroid | 56 | 16.7 | 3 | 0.9 | 21 | 6.3 | 32 | 9.6 |
| Vascular | 33 | 9.9 | 0 | 0.0 | 15 | 4.5 | 18 | 5.4 |
| Urogenital | 20 | 6.0 | 1 | 0.3 | 8 | 2.4 | 11 | 3.3 |
| Cardiac | 2 | 0.6 | 0 | 0.0 | 0 | 0.0 | 2 | 0.6 |
| Breast | 1 | 0.3 | 0 | 0.0 | 0 | 0.0 | 1 | 0.3 |
