# Supplementary material for: Survey on the current usage of ultrasound-guided procedures in Korean Medicine Clinics and Hospitals
Source: Medicine (Baltimore). 2024 Apr 5;103(14):e37659. doi: 10.1097/MD.0000000000037659 (PMC10994457; doi:10.1097/MD.0000000000037659)
Supplement: Supplementary file 8 [file medi-103-e37659-s008.docx]

**Supplementary Table 8.** KMDs’ experience concerning safety, effectiveness, expertise, and patient satisfaction related to the use of ultrasound guidance

| Q. Compared to the cases of unguided interventions, how much improvement in safety have you experienced with the use of ultrasound guidance? | | | | | |
| --- | --- | --- | --- | --- | --- |
|  | Not improved | Almost not improved | Slightly improved | Much improved | Very much improved |
| N | 5 | 15 | 65 | 143 | 107 |
| % | 1.5 | 4.5 | 19.4 | 42.7 | 31.9 |
| Q. Compared to the cases of unguided interventions, how much improvement in the effectiveness of a KM intervention have you experienced with the use of ultrasound guidance? | | | | | |
|  | Not improved | Almost not improved | Slightly improved | Much improved | Very much improved |
| N | 4 | 16 | 102 | 115 | 98 |
| % | 1.2 | 4.8 | 30.4 | 34.3 | 29.3 |
| Q. Compared to the cases of unguided interventions, how much improvement in expertise as a health professional have you experienced with the use of ultrasound guidance? | | | | | |
|  | Not improved | Almost not improved | Slightly improved | Much improved | Very much improved |
| N | 2 | 7 | 38 | 125 | 163 |
| % | 0.6 | 2.1 | 11.3 | 37.3 | 48.7 |
| Q. Compared to the cases of unguided interventions, how much improvement in patient satisfaction have you experienced with the use of ultrasound guidance? | | | | | |
|  | Not improved | Almost not improved | Slightly improved | Much improved | Very much improved |
| N | 2 | 11 | 67 | 140 | 115 |
| % | 0.6 | 3.3 | 20.0 | 41.8 | 34.3 |

KMDs, doctors of Korean medicin
