# Supplementary material for: Survey on the current usage of ultrasound-guided procedures in Korean Medicine Clinics and Hospitals
Source: Medicine (Baltimore). 2024 Apr 5;103(14):e37659. doi: 10.1097/MD.0000000000037659 (PMC10994457; doi:10.1097/MD.0000000000037659)
Supplement: Supplementary file 9 [file medi-103-e37659-s009.docx]

**Supplementary Table 9.** Opinions on NHI reimbursement of ultrasound-guided intervention and pricing of the fee for the service

| Q. Do you agree on the necessity of NHI reimbursement of ultrasound-guided KM intervention? | | | | | | | | | | | |
| --- | --- | --- | --- | --- | --- | --- | --- | --- | --- | --- | --- |
|  | Strongly Agree | | Agree | | Undecided | | | Disagree | | Strongly Disagree | |
| N | 280 | | 38 | | 10 | | | 7 | | 0 | |
| % | 83.6 | | 11.3 | | 3.0 | | | 2.1 | | 0.0 | |
| Q. What would be the appropriate price for the fee of “ultrasound-guided acupuncture” for treating shoulder joints? | | | | | | | | | | | |
|  | USD ≤7.66 | | USD 7.66-22.97 | | USD 22.97 - 38.28 | | | USD 38.28 – 53.60 | | USD 53.60 - 76.57 | |
|  | KRW ≤10,000 | | KRW 10,000–30,000 | | KRW 30,000–50,000 | | | KRW 50,000–70,000 | | KRW 70,000–100,000 | |
| N | 33 | | 133 | | 102 | | | 57 | | 10 | |
| % | 9.9 | | 39.7 | | 30.4 | | | 17.0 | | 3.0 | |
| Q. What would be the appropriate price for the fee of “ultrasound-guided pharmacopuncture” for treating shoulder joints | | | | | | | | | | | |
|  | USD ≤7.66 | USD 7.66-22.97 | | USD 22.97 - 38.28 | | USD 38.28 – 53.60 | USD 53.60 - 76.57 | | USD ≥ 76.57 | | Do not know |
|  | KRW ≤10,000 | KRW 10,000–30,000 | | KRW 30,000–50,000 | | KRW 50,000–70,000 | KRW 70,000–100,000 | | KRW  ≥100,000 | |  |
| N | 8 | 40 | | 105 | | 81 | 49 | | 48 | | 4 |
| % | 2.4 | 11.9 | | 31.3 | | 24.2 | 14.6 | | 14.3 | | 1.2 |
| Q. What would be the appropriate price for the fee of an “ultrasound-guided acupotomy” service for treating shoulder joints? | | | | | | | | | | | |
|  | USD ≤7.66 | USD 7.66-22.97 | | USD 22.97 - 38.28 | | USD 38.28 – 53.60 | USD 53.60 - 76.57 | | USD ≥ 76.57 | | Do not know |
|  | KRW ≤10,000 | KRW 10,000–30,000 | | KRW 30,000–50,000 | | KRW 50,000–70,000 | KRW 70,000–100,000 | | KRW  ≥100,000 | |  |
| N | 6 | 45 | | 83 | | 69 | 36 | | 77 | | 19 |
| % | 1.8 | 13.4 | | 24.8 | | 20.6 | 10.7 | | 23.0 | | 5.7 |

KM, Korean medicine; NHI, National Health Insurance
The exchange rate was applied at 1 USD to 1,306.05 KRW(1 st Dec, 2023).
